# Extraocular muscle repositioning as the last therapeutic option for a patient with a severe course of Graves’ Ophthalmopathy: a case report

**DOI:** 10.1186/s12886-018-0718-1

**Published:** 2018-02-27

**Authors:** Andrea Rau, Matthias Klopfer, Niklas Rommel, Mechthild Rau-Fornefeld, Andreas Kolk

**Affiliations:** 1 Department of Oral and Maxillofacial Surgery, Klinikum rechts der Isar, Technische Universität München, Ismaningerstr. 22, D-81675 Munich, Germany; 2 Department of Ophthalmology, Klinikum rechts der Isar, Technische Universität München, Ismaningerstr. 22, D-81675 Munich, Germany; 3 Bochum, Germany

**Keywords:** Graves’ Ophthalmopathy, Graves’ disease, Loss of vision, Orbital decompression, Strabismus, Case report

## Abstract

**Background:**

Graves’ disease is a common autoimmune inflammatory condition of the thyroid. About one in four of affected patients also develop orbital symptoms like exophthalmos, proptosis and diplopia - called Graves’ Ophthalmopathy. Not all patients respond well to the standard therapy of systemic glucocorticoid administration. The inflammatory swelling of the intraorbital muscles can lead to pressure-induced damage of the optic nerve.

Orbital decompression surgery is a therapeutic option for these patients with varying success. Other symptoms like the extreme malposition of the ocular globe are poorly addressed by decompression surgery and demand for different therapeutic approaches.

**Case presentation:**

Presented is the case of a 46-year old patient with an acute exacerbation of Graves’ ophthalmopathy. Clinically apparent was a convergent strabismus fixus with severe hypotropia of both eyes. The patient suffered from attacks of heavy retrobulbar pain and eyesight deteriorated dramatically. Since neither systemic glucocorticoid therapy nor orbital decompression surgery had helped to halt the progress of the disease, a decision was made in favour of the surgical release and repositioning of the inferior and medial rectus muscle as a final therapeutic option. Surgery of both eyes was performed consecutively within one week. Detailed descriptions and illustrations of the surgical steps and treatment outcome are provided supplemented by a discussion of the current literature.

**Conclusions:**

Graves’ Ophthalmopathy is a variant and therapeutically challenging disease. Exceptional courses of the disease call for therapeutic approaches off the beaten track. Surgical extraocular muscle repositioning, which has not been described before in the context of Graves’ Ophthalmopathy, proved to be effective in improving the patient’s eyesight and quality of life. Furthermore, we regard the measurement of extraocular muscle volume as a valuable method to monitor the course of Graves’ Ophthalmopathy.

## Background

Graves’ ophthalmopathy (GO), an autoimmune disease of the retroorbital tissue, is clinically relevant in 20–25% cases of Graves’ disease [1]. Patients with GO are more likely to be women by a 2:1 ratio [2]. The disease has a very variant course and often aggravates after thyroidectomy or radioiodine therapy, although more recent studies query this correlation [3, 4]. Smokers run the highest risk of worsening of GO [5]. The pathogenesis and interaction of the causal factors in GO is still not clearly understood, which makes GO therapy challenging and often frustrating. Ocular irritation, swelling of eyelids, disfiguring proptosis and diplopia are common symptoms of GO, which come along with severe emotional distress and significantly reduced life quality for the patients [6]. In serious courses of the disease, even total loss of vision may result. To prevent these dramatic consequences, a close interdisciplinary collaboration between endocrinologists, ophthalmologists and surgeons is required [7]. When steroid immunosuppression fails to halt the progress of GO, there is only a very limited number of further therapeutic options. Based on the hypothesis, that B cell depletion may be beneficial to GO, the systemic application of the anti-CD20 monoclonal antibody Rituximab has been promising in a recently published controlled study [8]. Three wall orbital decompression via various surgical techniques is a common treatment modality used in advanced stages of GO to prevent complete vision loss due to local pressure. However, a current review of the literature reveals a lack of controlled studies investigating the effectiveness and complications of decompressive surgery [9]. Furthermore, decompressive surgery itself was also found to provoke GO in rare cases, called “delayed decompression-related reactivation” [10].

## Case presentation

**Fig. 1:**
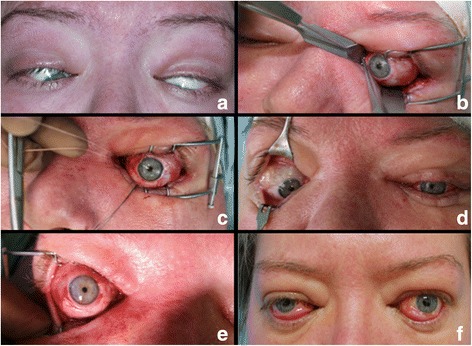
**a** Clinical appearance of the patient before the operation: extreme eso- and hypotropia of both eyes. **b** Identification of the inferior rectus muscle and wrapping with a squint hook. **c** Corrected position of the left eye after release of the inferior rectus muscle and the medial rectus muscle. **d** Clinical appearance of the patient one week after surgery of the left eye, before surgery of the right eye. **e** Corrected position of the right eye after release of the inferior rectus muscle and the medial rectus muscle. **f** Clinical appearance of the patient three months after surgery

**Fig. 2:**
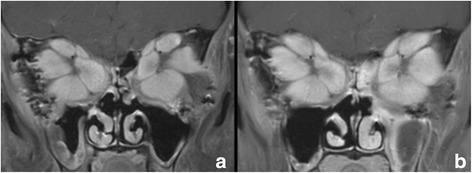
Orbital MRI Scan. Contrast enhanced (Magnevist®), coronal, fat-suppressed (Philips 3 T, Ingenia) 2D TSE sequence. Acquisition parameter: TE 10 ms, TR 611 ms, ETL 4, matrix 360 × 291, slice thickness 3 mm. (**a**) preoperative (**b**) three months after surgery

A 46-year old female patient, who had been suffering from autoimmune thyroid disease for eight years, presented at our clinic with an acute exacerbation of GO. Clinical examination revealed a convergent strabismus fixus with severe hypotropia of both eyes (Fig. 1a). The patient complained of increasing loss of eyesight and heavy retrobulbar pain. Visual acuity had deteriorated significantly from 0.6/0.5 to 0.1/0.1 within 3 months. A contrast enhanced orbital MRI scan showed distinct swelling of all extraocular muscles with bilateral compression of the optic nerve (Fig. 2a). There was no history of comorbidities except nicotine abuse. Laboratory tests showed a euthyroid biochemical status with TSH within the normal range, but elevated levels of Anti-Thyroid Peroxidase Antibody, Anti-Thyroglobulin Antibody and Thyroid Receptor Antibody. The patient’s daily medication comprised of 200 μg L-Thyroxin and 200 μg Selenium. Over many years, the patient had shown only mild to moderate symptoms of GO, but following a thyroidectomy, the symptoms had recently worsened dramatically. Since the disease could not be controlled by high-dose systemic glucocorticoid therapy, bilateral three wall orbital decompression had been performed twice previously. In the first step, the medial orbital wall had been resected via an endonasal approach. Due to ongoing findings as before, two months later partial resection of the orbital floor and fenestration of the lateral orbital wall via a combined transconjunctival/transcaruncular approach with piezosurgery had been performed. In addition, high-dose systemic glucocorticoid therapy was conducted prior to surgery and for the first two months after surgery. Glucocorticoid medication had to be gradually reduced until zero because the patient suffered from an upcoming depression and Cushing syndrome. Orbital radiotherapy for the treatment of thyroid eye disease had been considered as a therapeutic option, but the rapid progress of the disease with the growing risk of dysthyroid optic neuropathy forced us to act more quickly than orbital radiotherapy could perform [11]. Since the patient increasingly suffered from loss of vision and heavy pain attacks because of medial caudal squinting, we decided to correct the hypo- and esotropia surgically by releasing and repositioning the insertion points of the inferior and medial rectus muscle. Acute surgery was the last remaining treatment option.

### Surgical procedures

Operations on the left and right eye were performed consecutively with an interval of one week. The eye with the lower vision (left side) was chosen first. A forced duction test showed a complete fixation of the bulb. We opted for a transconjunctival approach (limbal incision) combined with lateral canthotomy to gain access to the dorsal part of the inferior part of the eye bulb. The insertion of the inferior rectus muscle was localized and then circuited with a squint hook (Fig. 1b). A non-absorbable polyethylene suture (Mersilene 4.0, Ethicon, U.S.) was placed at the anterior rim of the muscle before the muscle was detached from the outer bulb. This release in tension immediately resulted in the spontaneous elevation of the bulb. Lengthening of the muscle with an interposition graft (e.g. fascia lata) was not possible because of the deep retraction of the muscle. Instead, the polyethylene thread loop was directly fixed to the sclera at the former muscle insertion area, placing the muscle 12-15 mm dorsally with regard to its original fixation position. By marking of the original muscle insertion point with a non-absorbable polyethylene suture, an option could be preserved for a more precise muscle readaption at a later stage. Subsequently, the same procedure was carried out with the medial rectus muscle. Less tension allowed direct refixation of the muscle to the sclera without bridging by the polyethylene suture. The bulb was freely movable and remained vertically and horizontally in a primary position (Fig. 1c). Despite a significant exophthalmos, passive eyelid closure could easily be performed. The significant conjunctival contraction caused by the long-term hypotropia meant that only partial conjunctival wound closure with a polyglactin suture (Vicryl 7.0, Ethicon, U.S.) was possible. No postoperative complications occurred under the postoperative systemic antibiotic medication with Clindamycin of 3x600mg per day over three days supplemented by local application of Neomycin eye ointment for one week. Surgery of the right eye was conducted in the same manner (Fig. 1d and e) and under the same perioperative protocol one week later. Additionally, two mucosal grafts of 3.0 × 1.5 cm were harvested bilaterally from the inner cheek to be used for the bilateral reconstruction of the conjunctiva. The intraoral donor sites were closed primarily by using Vicryl 3.0 (Ethicon, U.S.). Starting from extreme eso- and hypotropia, the operation succeeded in repositioning the bulbs into the vertical and horizontal primary position with no restriction of passive movements. Three months postoperatively, the patient was free of pain and had a visual acuity of 0.3/0.6. Visual field testing (Goldmann perimetry) showed only slight concentric bilateral restrictions. Surprisingly, the patient did not suffer from diplopia despite the persistent restriction of active ocular mobility and a moderate bilateral exotropia (Fig. 1f).

Follow-up examinations of the patient will be performed at close intervals, including ophthalmological check-ups and the testing of thyroid blood parameters. Contrast enhanced orbital MRI scans will allow the measurement of extraocular muscle volume, as described by Kolk et al. [12]. Orbital MRI scans were performed preoperatively and three months postoperatively (Fig. 2a and b) and the volumes of the extraocular muscles were calculated by using manual segmentation (Osirix Imaging software 5.9) (Table 1). During this time interval, the total extraocular muscle volume increased from 24.91cm^3^ to 29.29cm^3^. Together with the ongoing high levels of thyroid-specific antibodies (Anti-Thyroid Peroxidase Antibody, Anti-Thyroglobulin Antibody and Thyroid Receptor Antibody), this indicated that the patient was still in an active stage of GO. Volumetric measurements of the extraocular muscles, based on follow-up MRI scans, will help to monitor the course of the disease. Further squint surgery, in terms of a precise readaption of the extraocular muscles, will be postponed to the future, when a more stable stage of the systemic autoimmune disease will be reached.

**Table 1:** Results of pre- and postoperative measurements of the extraocular muscle volume: Lev (levator palpebrae superioris), SR (superior rectus), SO (superior oblique), MR (medial rectus), IR (inferior rectus), LR (lateral rectus), IO (inferior oblique)

	Preoperative Volume (cm^3^)	Postoperative Volume (cm^3^)	Relative Change of Volume (%)
Muscle right eye			
Lev	0.5	1.53	306
SR	2.15	1.91	89
SO	0.8	0.87	109
MR	3.76	3.77	100
IR	3.08	3.32	108
LR	2.29	3.22	141
IO	0.31	0.33	106
Total right eye	12.89	14.95	116
Muscle left eye			
Lev	0.65	0.98	151
SR	1.98	2.53	128
SO	0.85	0.81	95
MR	2.72	3.39	125
IR	2.91	3.18	109
LR	2.61	3.08	118
IO	0.3	0.37	123
Total left eye	12.02	14.34	119
Total both eyes	24.91	29.29	118

## Discussion

Decisions of indications for surgical intervention in GO have to be performed based on the activity classification of GO by using clinical activity scores (CAS). The CAS is based on a points system and includes pain or pressure having a periorbital or retro-orbital distribution, with eye movements, signs of swelling, redness of eyelids, chemosis, inflammation, proptosis > 2 mm, decreased eye movement > 80% and decreased visual activity over 1–3 months [13].

GO rarely requires surgical intervention in the acute stage of the disease. This applies only to those cases that do not respond to steroids or radiation and have progressive compression of the optic nerve or refractory corneal ulceration. In most cases, GO is operated on at the chronic stage for reasons of corneal protection, retrobulbar pain or increasing intra-orbital pressure. In addition, psychosocial or cosmetic reasons might also confirm the indication.

A distinction is made between two operative principles: decompression by volume expansion of the bony orbital walls or orbital volume reduction by fat resection. Numerous operative access routes have been described; in the current case, a transconjunctival approach was chosen, which, in addition to the overview, also enabled fat resection, in conjunction with decompression with the greatest possible tissue preservation. A sole fat resection would not have been sufficient in this patient and is generally not very efficient, especially in orbital muscle thickening as in the case described.

Normally, about 3 to 6 months after decompression of the orbital walls, orbital muscle corrective surgery is performed. At the same time, eyelid retraction surgery is necessary for persistent lid retraction. In the current case, these measures could not be undertaken so far, since the activity change of GO is ongoing and, accordingly, the eye muscle thickening is still pronounced.

## Conclusions

The described procedure is definitely not the first choice method. However, after the failure of all conventional therapeutic measures, extraocular muscle repositioning helped, in this case, to prevent acute loss of vision and tremendously improved the patient’s life quality. Furthermore, we regard the measurement of the extraocular muscle volume as a valuable tool for monitoring the course of the disease and the timing of treatment steps.

## Abbreviations

ETL: Echo-Train Length
GO: Graves’ Ophthalmopathy
MRI: Magnetic Resonance Imaging
TE: Echo Time
TR: Repetition Time
TSE: Turbo Spin Echo
TSH: Thyroid-Stimulating Hormone
